# Efficient gradient computation for dynamical models

**DOI:** 10.1016/j.neuroimage.2014.04.040

**Published:** 2014-09

**Authors:** B. Sengupta, K.J. Friston, W.D. Penny

**Affiliations:** Wellcome Trust Centre for Neuroimaging, Institute of Neurology, University College London, 12 Queen Square, London WC1N 3BG, UK

**Keywords:** Augmented Lagrangian, Adjoint methods, Dynamical systems, Dynamic causal modelling, Model fitting

## Abstract

Data assimilation is a fundamental issue that arises across many scales in neuroscience — ranging from the study of single neurons using single electrode recordings to the interaction of thousands of neurons using fMRI. Data assimilation involves inverting a generative model that can not only explain observed data but also generate predictions. Typically, the model is inverted or fitted using conventional tools of (convex) optimization that invariably extremise some functional — norms, minimum descriptive length, variational free energy, etc. Generally, optimisation rests on evaluating the local gradients of the functional to be optimized. In this paper, we compare three different gradient estimation techniques that could be used for extremising any functional in time — (i) finite differences, (ii) forward sensitivities and a method based on (iii) the adjoint of the dynamical system. We demonstrate that the first-order gradients of a dynamical system, linear or non-linear, can be computed most efficiently using the adjoint method. This is particularly true for systems where the number of parameters is greater than the number of states. For such systems, integrating several sensitivity equations – as required with forward sensitivities – proves to be most expensive, while finite-difference approximations have an intermediate efficiency. In the context of neuroimaging, adjoint based inversion of dynamical causal models (DCMs) can, in principle, enable the study of models with large numbers of nodes and parameters.

## Highlights

• We compare three methods to compute gradients in dynamical systems.
• The methods are finite-differences, forward sensitivity and reverse adjoints.
• The adjoint method is 50–70 folds more efficient.

## Introduction

An important goal of systems neuroscience is to integrate empirical data from various neuroimaging modalities with biologically informed models that describe the underlying generative processes. Here, the data to be explained are for example M/EEG and fMRI recordings made while subjects perform various experimental tasks, and the underlying neurodynamic processes are framed in terms of differential equations describing activity in neural masses, mean fields, or neural fields (David et al., 2006, Deco et al., 2008, Friston et al., 2003).

Considerable insight can be gained from studying the emergent properties of such neurodynamic processes. These can then be qualitatively compared with empirical data, allowing consilience among multiple levels of description (Gazzaniga, 2010, Hopfield and Brody, 2001, Wilson, 1999). An alternative approach is to directly fit neurodynamical models to neuroimaging data using standard model fitting procedures from statistics and machine learning (Bishop, 2006, Press et al., 1992). Differences in the generative processes induced by experimental manipulations can then be associated with changes in underlying brain connectivity. One example of such an approach is Dynamic Causal Modelling (DCM) (Friston et al., 2003) which fits differential equation models to neuroimaging data using a variational Bayesian scheme (Friston et al., 2007).

More generally, in the statistics and machine learning literature various methods have been employed to fit differential equations to data, from maximum likelihood approaches (Ramsay et al., 2007) to Bayesian sampling algorithms (Calderhead and Girolami, 2009, Vyshemirsky and Girolami, 2008). The majority of these convex optimisation approaches involve computing the gradient; the change in the cost function produced by a change in model parameters. This gradient is then combined with information from line searches (e.g., Wolfe's conditions) or methods involving a Newton, quasi-Newton (low-rank) or Fisher information based curvature estimators to update model parameters (Bishop, 1995, Nocedal and Wright, 2006, Press et al., 1992). The main computational bottleneck in these algorithms is the computation of the gradient (or the curvature) of the parametric cost function. This motivates the search for efficient methods to evaluate gradients.

This paper compares three different methods for computing gradients, and studies the conditions under which each is preferred. The first is the Finite Difference (FD) method, which is the simplest and most general method — and is currently used in DCM. The second is the Forward Sensitivity (FS; also known as tangent linear) method, which has previously been proposed in the context of modeling fMRI time series (Deneux and Faugeras, 2006). The third is the Adjoint Method (AM) which has previously been used in the context of dynamical systems theory (Wang, 2013), weather forecasting (Errico, 1997), image registration (Clark, 2011) and single-neuron biophysics (Stemmler et al., 2012).

The paper is structured as follows — the methods section describes each approach including a mathematical derivation of the adjoint method. Examples of the FS and AM updates are then provided for the case of simple Euler integration. The results section reports numerical simulations that disclose the scaling characteristics of each method. Simulations are provided for linear dynamical and weakly-coupled oscillator systems. We conclude with a discussion of the relative merits of each method.

## Methods

We consider dynamical systems of the form

(1) $$\begin{array}{c} \dot{x}=f\left(x,p\right)\\ j\left(x,p\right)=-\frac{1}{2}{\left\|y-g\left(x,p\right)\right\|}^{2} \end{array}$$

where *x* is a state variable, the dot notation denotes a time derivative $\frac{\mathrm{dx}}{\mathrm{dt}}$, *t* is time, *f*(·) is the flow equation (dynamics), and *p* are model parameters. The model produces a prediction via an observation function *g* (*x*, *p*) and an instantaneous cost function *j* (*x*, *p*) measures the squared difference from data points *y*. The total cost is then given by the integral up to time point *T*

(2) $$J\left(p\right)=\int_{0}^{T}j\left(x,p\right)\mathrm{dt}.$$

We consider three methods for computing the gradient $\frac{\mathrm{dJ}}{\mathrm{dp}}$.

### Finite difference method

The (one-sided) finite difference approximation to the gradient is then

(3) $$\frac{\mathrm{dJ}}{dp_{i}}=\frac{J\left(p+\delta_{i}\right)-J\left(p\right)}{\delta_{i}}$$

where *δ_i_* denotes a small change (generally, $\sqrt{\epsilon }$ where ϵ is the machine epsilon) to the *i*th parameter. The error in the computation of this gradient is of order *δ_i_*. The computation of $\frac{\mathrm{dJ}}{\mathrm{dp}}$ requires *P* + 1 runs of the integration process, one for each model parameter. It is also possible to use central differences

(4) $$\frac{\mathrm{dJ}}{dp_{i}}=\frac{J\left(p+\delta_{i}\right)-J\left(p-\delta_{i}\right)}{2\delta_{i}}$$

which has an error of order *δ*_*i*_^2^ but requires 2*P* + 1 runs of the integration process. Variations on the vanilla FD approach are discussed in (Press et al., 1992, Richtmeyer and Morton, 1967).

### Forward Sensitivity method

The original dynamical model (Eq. (1)) can be implicitly differentiated w.r.t parameters to give

(5) $$\frac{d\dot{x}}{\mathrm{dp}}=\frac{\partial f}{\partial x}\frac{\mathrm{dx}}{\mathrm{dp}}+\frac{\partial f}{\partial p}.$$

If the state variables are of dimension *D* and the parameters of dimension *P* then the quantity $\frac{d\dot{x}}{\mathrm{dp}}$ is a *D* × *P* matrix, which can be vectorized to form a new flow function. This forms a new dynamical system of dimension *D* × *P* that can then be integrated using any numerical method to produce $\frac{\mathrm{dx}}{\mathrm{dp}}$ as a function of time. The Forward Sensitivity approach has been known since the publication of Gronwall's theorem (Gronwall, 1919). The cost gradient is then given by accumulating the sensitivity derivative $\frac{\mathrm{dx}}{\mathrm{dp}}$ over time according to:

(6) $$\begin{array}{l} \frac{\mathrm{dJ}}{\mathrm{dp}}=\int_{0}^{T}\frac{\mathrm{dj}}{\mathrm{dp}}\mathrm{dt}\\ \frac{\mathrm{dj}}{\mathrm{dp}}=\frac{\partial j}{\partial x}\frac{\mathrm{dx}}{\mathrm{dp}}+\frac{\partial j}{\partial p}\\ \;=\frac{\partial j}{\partial g}\frac{\partial g}{\partial x}\frac{\mathrm{dx}}{\mathrm{dp}}+\frac{\partial j}{\partial g}\frac{\partial g}{\partial p}. \end{array}$$

### Euler example

This section illustrates the FS approach first-order Euler integration of the dynamics

(7) $$x_{n}=x_{n-1}+\mathrm{\tau f}\left(x_{n-1},p\right)$$

at discrete times *t*(*n*). The FS method is based on differentiating this equation to give

(8) $$\frac{dx_{n}}{\mathrm{dp}}=\frac{dx_{n-1}}{\mathrm{dp}}+\tau \left[\frac{\partial f}{\partial x_{n-1}}\frac{dx_{n-1}}{\mathrm{dp}}+\frac{\partial f}{\partial p}\right].$$

**Fig. 1:**
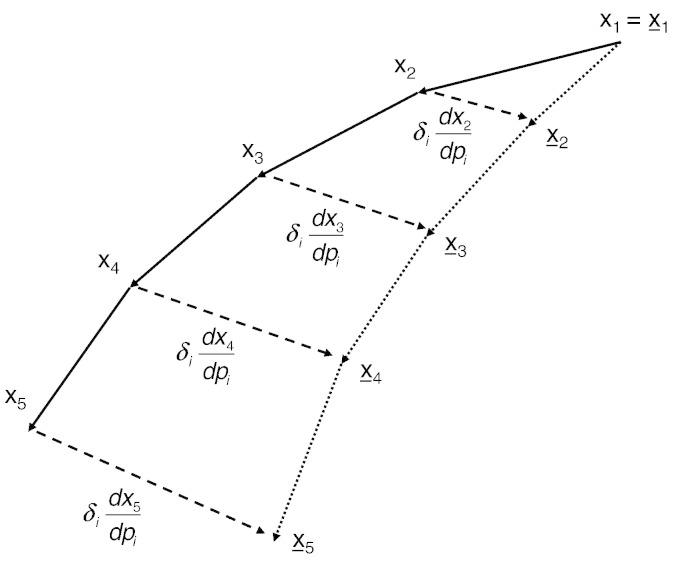
Forward Sensitivity The solid path indicates a trajectory of points *x*_*n*_, with *n* = 1…5, for a dynamical system with parameters p. The dotted path indicates the trajectory ${\underline{x}}_{n}$ for the same dynamical system but with parameters $\underline{p}=p+\delta_{i}$. The dotted path can be reached from the solid path via the total derivative $\frac{dx_{n}}{\mathrm{dp}}$. The Forward Sensitivity approach provides a method for computing this derivative.

This method is illustrated in Fig. 1 where the solid path indicates a trajectory of points *x*_*n*_ for a dynamical system with parameters *p* and the dotted path indicates the trajectory ${\underline{x}}_{n}$ for the same dynamical system but with parameters $\underline{p}=p+\delta_{i}$. The dotted path can be obtained from the solid path via the total derivative $\frac{dx_{n}}{dp_{i}}$ in the direction of the perturbation, *δ_i_*. The FS method provides a method for computing this derivative. Under a first order Euler approach for integrating the dynamics, this is implemented using the above recursion.

Because the perturbed path (dotted in Fig. 1) can be reached from the original trajectory via the total derivative $\frac{dx_{n}}{\mathrm{dp}}$, there is no need to separately integrate the system with parameters $\underline{p}$. Geometrically, the points ${\underline{x}}_{n}$ in Fig. 1 can be reached via the solid and dashed lines (rather than the dotted lines).

We rewrite the recursion equation as

(9) $$S_{x}\left(n\right)=S_{x}\left(n-1\right)+\tau \left[F_{x}\left(n-1\right)S_{x}\left(n-1\right)+F_{p}\left(n-1\right)\right]$$

where

(10) $$\begin{array}{l} F_{p}\left(n\right)={\left(\frac{\partial f}{\partial p}\right|}_{x_{n}}\\ F_{x}\left(n\right)={\left(\frac{\partial f}{\partial x}\right|}_{x_{n}}\\ S_{x}\left(n\right)={\left(\frac{\mathrm{dx}}{\mathrm{dp}}\right|}_{x_{n}.} \end{array}$$

*S_x_* is a [D × P] matrix, *F_x_* is [D × D] and *F_p_* is [D × P]. We then have

(11) $$\begin{array}{l} \frac{\mathrm{dJ}}{\mathrm{dp}}=\sum_{n=1}^{N}\frac{\partial j}{\partial x_{n}}\frac{dx_{n}}{\mathrm{dp}}\\ \;=\sum_{n=1}^{N}j_{x}\left(n\right)S_{x}\left(n\right) \end{array}$$

and *j_x_* (*n*) is the derivative of *j* (*x*, *p*) with respect to *x*, evaluated at *x_n_*. This method requires the derivatives *F_x_* and *F_p_*. These will be specific to the dynamical model in question and, in this paper, are computed analytically. We provide the Euler example here as a simple illustration of the method. The numerical simulations in this paper use a more accurate integration method (see below).

### Adjoint method

Errico (1997) and Giles and Pierce (2000) provide introductions to the adjoint method. A derivation of the adjoint method for dynamical models is provided rigorously in Cao et al. (2003) and in Hindmarsh and Serban (2002). Here, we provide an informal derivation, starting with the cost function

(12) $$J\left(p\right)=\int_{0}^{T}j\left(x,p\right)\mathrm{dt}.$$

The constraints implied by the dynamics allow us to write the Lagrangian

(13) $$L\left(p\right)=\int_{0}^{T}j\left(x,p\right)\mathrm{dt}+\int_{0}^{T}\lambda^{T}\left[\dot{x}-f\left(x,p\right)\right]\mathrm{dt}.$$

Once the system has been integrated (solved for *x*) we have $\dot{x}=f\left(x,p\right)$. Hence the second term in the Lagrangian disappears and we have

(14) $$\frac{\mathrm{dJ}}{\mathrm{dp}}=\frac{dL}{\mathrm{dp}}.$$

This is the gradient we wish to compute. So far it may seem that we have made no progress but it turns out that $\frac{dL}{\mathrm{dp}}$ can be computed efficiently.

Before proceeding further, we summarize the main ideas behind the adjoint method. The central concept is that the Lagrange vector *λ*^*T*^ constrains the dynamical system to variations around the forward path *x_n_*. The Lagrange vectors are of the same dimension as *x* and form a time series. Algebraically, the contribution of the total derivative $\frac{\mathrm{dx}}{\mathrm{dp}}$ to the gradient $\frac{\mathrm{dJ}}{\mathrm{dp}}$ is made zero, by setting *λ*^*T*^ appropriately. This means that the sensitivity derivative need not be calculated, resulting in a large computational saving. Instead, the gradient $\frac{\mathrm{dJ}}{\mathrm{dp}}$ can be expressed as a function of *λ*^*T*^. We will now go through this in a bit more detail:

The proof proceeds by differentiating Eq. (13) to give the gradient

(15) $$\frac{\mathrm{dJ}}{\mathrm{dp}}=\int_{0}^{T}\left(\frac{\partial j}{\partial x}\frac{\mathrm{dx}}{\mathrm{dp}}+\frac{\partial j}{\partial p}\right)\mathrm{dt}+\int_{0}^{T}\lambda^{T}\left(\frac{d\dot{x}}{\mathrm{dp}}-\frac{\partial f}{\partial x}\frac{\mathrm{dx}}{\mathrm{dp}}-\frac{\partial f}{\partial p}\right)\mathrm{dt}.$$

The term involving the change in total derivative, $\frac{d\dot{x}}{\mathrm{dp}}$, can be rewritten using integration by parts

(16) $$\int_{0}^{T}\lambda^{T}\frac{d\dot{x}}{\mathrm{dp}}\mathrm{dt}={\left[\lambda^{T}\frac{\mathrm{dx}}{\mathrm{dp}}\right]}_{0}^{T}-\int_{0}^{T}\frac{d\lambda^{T}}{\mathrm{dt}}\frac{\mathrm{dx}}{\mathrm{dp}}\mathrm{dt}.$$

Substituting this into the previous expression and rearranging to group together terms involving the sensitivity derivative $\frac{\mathrm{dx}}{\mathrm{dp}}$ give

(17) $$\begin{array}{l} \frac{\mathrm{dJ}}{\mathrm{dp}}=\int_{0}^{T}\frac{\mathrm{dx}}{\mathrm{dp}}\left(\frac{\partial j}{\partial x}-\lambda^{T}\frac{\partial f}{\partial x}-\frac{d\lambda^{T}}{\mathrm{dt}}\right)\mathrm{dt}\\ \;+\int_{0}^{T}\left(\frac{\partial j}{\partial p}-\lambda^{T}\frac{\partial f}{\partial p}\right)\mathrm{dt}+{\left[\lambda^{T}\frac{\mathrm{dx}}{\mathrm{dp}}\right]}_{0}^{T}. \end{array}$$

The adjoint vector *λ*^*T*^ can be used to eliminate the first term involving the sensitivity derivative. This term is zero when:

(18) $$\frac{d\lambda^{T}}{\mathrm{dt}}=\frac{\partial j}{\partial x}-\lambda^{T}\frac{\partial f}{\partial x}.$$

This is known as the adjoint equation and is used to compute *λ*^*T*^. The gradient is then given by

(19) $$\frac{\mathrm{dJ}}{\mathrm{dp}}=\int_{0}^{T}\left(\frac{\partial j}{\partial p}-\lambda^{T}\frac{\partial f}{\partial p}\right)\mathrm{dt}+{\left[\lambda^{T}\frac{\mathrm{dx}}{\mathrm{dp}}\right]}_{0}^{T}.$$

As our goal has been to avoid computation of the sensitivity derivative, $\frac{\mathrm{dx}}{\mathrm{dp}}$, we can eliminate the last term above by integrating the adjoint equations backward in time, starting with λ*_T_* = *0*. The starting value for the adjoint equation is arbitrary and it can be proven that if ${\left(\lambda \right|}_{t=t_{f,a}}$ and ${\left(\lambda \right|}_{t=t_{f,b}}$ are two different starting values for the adjoint equation with solutions *λ*_*a*_ and *λ*_*b*_ respectively, then ${\left(\frac{\mathrm{dJ}}{\mathrm{dp}}\right|}_{\lambda_{a}}={\left(\frac{\mathrm{dJ}}{\mathrm{dp}}\right|}_{\lambda_{b}}$. Therefore, there exist infinitely many starting conditions for the adjoint equation that yields the same parametric gradient.

If the initial conditions do not depend on the parameters, as we assume for our numerical examples, then we have $\frac{\mathrm{dx}}{\mathrm{dp}}=0$ at *t* = 0 and the gradient reduces to

(20) $$\frac{\mathrm{dJ}}{\mathrm{dp}}=\int_{0}^{T}\left(\frac{\partial j}{\partial p}-\lambda^{T}\frac{\partial f}{\partial p}\right)\mathrm{dt}.$$

This equality can now be used to compute the parametric gradients, given the backwards solution of the adjoint equation.

There are no restrictions on the functional form to make the adjoint method viable — if one can pose the optimization problem via a Lagrangian, then the adjoint method could be used for any dynamical system (ordinary-, delay-, random- and partial-differential equation). The one and only constraint is the stability of the adjoint equation for the underlying dynamical system. Thus, static or dynamical systems that are routinely used in neuroimaging are amenable to an adjoint formulation under some loss-function including stochastic DCMs that have an analytical model for the noise (of class C^ω^). Table 1 highlights the key differences between all of the methods and the crucial steps required in each of them.

**Table 1:** Comparison of the different gradient computation methods. The flow eqn. is either linear or non-linear, with P parameters and N state variables.

	Finite differences	Forward sensitivities	Adjoint
Suitability	Arbitrary	N ≫ P	P ≫ N
Cost	(1 + *P*) flow eqns.	*P* non-linear sensitivity eqns. + 1 flow eqn.	1 linear adjoint eqn. + 1 flow eqn.
Key steps	1. Integrate flow eqn. 2. Parametrically perturb flow P times	1. Integrate the coupled flow and sensitivity eqns.	1. Integrate flow eqn. 2. Integrate adjoint eqn.

### Euler example

The specification of the adjoint method starts from the specification of the Lagrangian. For us, this has the particular form

(21) $$L=-\frac{1}{2}\sum_{n}||y_{n}-g\left(x_{n}\right)||{}^{2}+\sum_{n}\lambda_{n}\left[x_{n}-x_{n-1}-\mathrm{\tau f}\left(x_{n-1},p\right)\right]$$

where the first term is the original cost function, the second term enforces the constraint embodied in the Euler integration of the state dynamics, and *λ_n_* is a [1 × D] vector of Lagrange multipliers. Because L is a scalar, and the state *x_n_* is a column vector, the Lagrange multipliers must be a row vector. It is in this sense that they are adjoint (or transposed) to the states. The derivative of L with respect to the states is then given by

(22) $$\frac{dL}{dx_{n}}=g_{x}\left(n\right)\left[y_{n}-g\left(x_{n}\right)\right]+\lambda_{n}-\lambda_{n+1}-\tau \lambda_{n+1}F_{x}\left(n\right)$$

where g*_x_*(*n*) is the derivative of g (*x*, *p*) with respect to *x*, evaluated at *x*_*n*_. Setting Eq. (22) to zero (i.e., solving for the states) gives

(23) $$\lambda_{n}=\lambda_{n+1}\left[I+\tau F_{x}\left(n\right)\right]-g_{x}\left(n\right)\left[y_{n}-g\left(x_{n}\right)\right].$$

This is a backward recursion, known as the adjoint equation, that starts with *λ*_*N*_ = 0. After solving the adjoint equations we can enter *λ*_*n*_ into Eq. (20), giving

(24) $$\frac{\mathrm{dJ}}{\mathrm{dp}}=\tau \sum_{n=1}^{N}\left[j_{p}\left(n\right)-\lambda_{n}F_{p}\left(n\right)\right]$$

where *j_p_* (*n*) is the derivative of *j* (*x*, *p*) with respect to *p*, evaluated at *x*_*n*_. If the observation function does not depend on model parameters then the first term disappears. A first order Euler Adjoint method has been used previously in the context of image registration (Clark, 2011). However, we provide the Euler example here as an illustration of the method. The numerical simulations in this paper use a more accurate integration method (see below).

### Stability

It is known that if flows are prescribed as ODEs, then their adjoint solutions are also stable (Cao et al., 2003). Under these conditions, the numerical stability of the adjoint system is guaranteed when the adjoint equation is integrated backwards in time, in the sense that the flow is reversed. Consider a linear autonomous system, *f* = A*x* + *B* where *A* ∈ ℝ^*n* × *n*^ and *B* ∈ ℝ^*n*^ and both are invariant in time. Being linear in states with pre-defined initial conditions, such a system can be analytically integrated to yield a solution as a sum of its *n* matrix exponentials with unique eigenvectors and their respective eigenvalues. Such a system is asymptotically stable when the eigenvalues have a negative real part i.e., Re(*Λ*(*A*)) < 0. For such a linear autonomous system the eigenvalues of the adjoint equation, $\dot{\lambda }=-\left(\frac{\mathrm{df}}{\mathrm{dx}}){}^{T}\cdot \lambda =-A^{T}\lambda \right)$ have a positive real part, proving to be asymptotically unstable. If one were now to reverse the flow i.e., ${\dot{\lambda }}_{*}={\left(\frac{\mathrm{df}}{\mathrm{dx}}\right)}^{T}\cdot \lambda_{*}=A^{T}\lambda_{*}$, the eigenvalues then have a negative real part and the dynamics is asymptotically stable. One can derive similar results for non-linear equations using a perturbation expansion, suggesting the condition of asymptotic and uniform stability is guaranteed when the adjoint equations are integrated backwards.

### Integration

For the numerical results in this paper, we used MATLAB's ode15s function which implements a variable order method for integrating stiff differential equations (Shampine and Reichelt, 1997). Two important parameters governing the operation of this algorithm are the absolute, *a*, and relative, *r*, error tolerances. The estimated error in each integration step is constrained to be less than *max*(*r*|*x*_*n*_|, *a*).

The absolute and relative tolerances were set to 10^− 7^ for each of the gradient computation methods although results were also obtained with different sets of tolerances, taking on values *a*_*FD*_, *a*_*FS*_, *a*_*AM*_ and *r*_*FD*_, *r*_*FS*_, *r*_*AM*_ for the Finite Difference, Forward Sensitivity and Adjoint Methods respectively. When tolerances were set differently, these values were tuned for each problem (linear/nonlinear) so as to achieve good agreement among the methods.

## Results

Custom MATLAB scripts were written to implement each of the gradient computation methods.

### Linear models

**Fig. 2:**
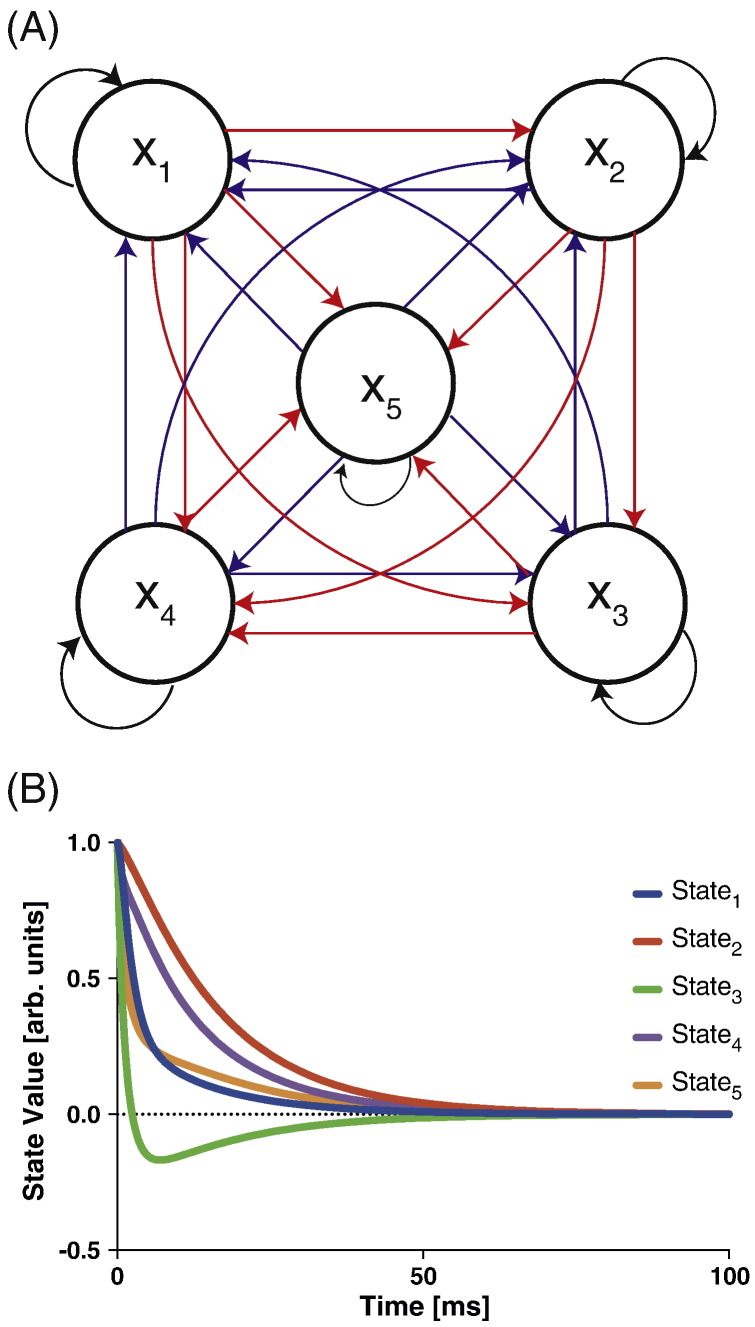
Linear System (A) The 5-dimensional state-space model and (B) the linear evolution of its eigenstates.

First we consider the linear models

(25) $$\dot{x}=\mathrm{Ax}$$

where *x* is a *D*-dimensional state vector with initial value *x*_0_ = 1, and *A* is a *D* × *D* connectivity matrix. Readers familiar with DCM for fMRI will recognize *A* as the endogenous or average connectivity matrix. A model with *D* states therefore has *P* = *D*^2^ parameters (Fig. 2(A)). The system is integrated from time 0 to *T*. To ensure stability, we constructed A using the linear expansion

(26) $$A=\sum_{d=1}^{D}q_{d}v_{d}v_{d}^{T}$$

where *v*_*d*_ ∼ N(*v*_*d*_; 0, 1) are standard *D*-dimensional Gaussian random vectors, which are serially orthogonalized. The scalars *q_d_* are negative real numbers so that the corresponding eigenstates are exponentially decaying modes. The values of *q_d_* were set so that the corresponding time constants were between *T*/5 and *T*. Fig. 2(B) shows the time series for five such eigenstates.

For each model dimension considered (see below) we generated a state trajectory using known model parameters generated as described above. We then created an observable data time series y*_n_* = *g*(*x_n_*) with the observation function *g*(*x_n_*) = *x_n_*, that is, all of the dynamical states are observed.

We then created ‘perturbed’ parameters by adding Gaussian noise with a standard deviation of 10% of the original parameters. The cost function was defined as

(27) $$J=-\frac{1}{2}\sum_{n}{\left[y_{n}-g\left(x_{n}\right)\right]}^{2}.$$

To summarize, the ‘data points’ *y_n_* were created using the original parameters and the ‘model predictions’, *g*(*x*_*n*_) used the perturbed parameters. Gradients were then estimated at this perturbed point.

**Fig. 3:**
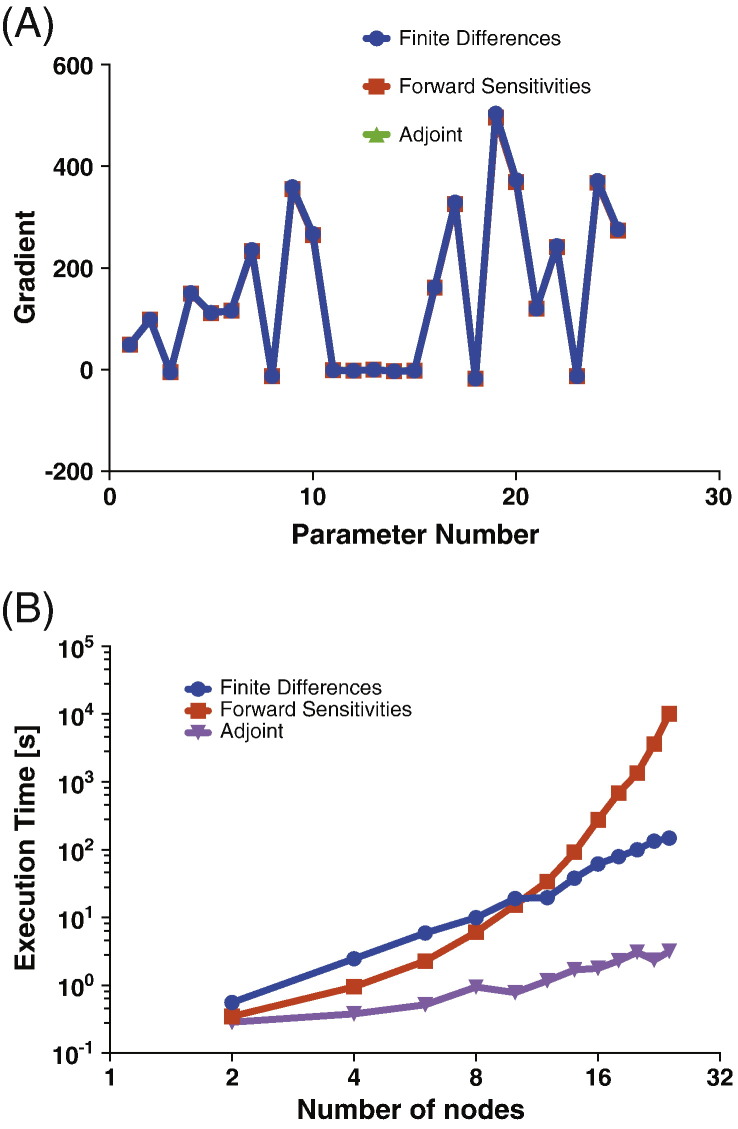
Computational efficiency for linear systems (A) Comparison of the parametric gradient obtained by the three methods. (B) Scaling of run-time as a function of the number of nodes. The absolute and relative tolerances of FD and FS methods were set to 10^− 7^ while the tolerances for the AM method were fixed to 10^− 3^. Simulation time was fixed at 400 ms.

The systems were integrated using the tolerances of FD and FS fixed at 10^− 7^. Although, the tolerance of AM was adjusted so as to achieve the best fit to the FD based gradient estimate, for the efficiency-scaling simulations we fixed it at a lower value of 10^− 3^. This is illustrated in Fig. 3(A) that shows the estimated gradients for a *D* = 5 dimensional linear system. Setting the tolerance of the AM method to 10^− 3^ did not affect the mean-squared deviation of the gradients obtained between the FD and the AM methods (data not shown).

We then compared the three gradient computation methods. Fig. 3(B) plots the computation time as a function of state dimension. For a 28-node system with 784 model parameters the computation time for the adjoint method is 77 times less than for the finite difference method.

### Nonlinear models

Next, we consider weakly coupled oscillators of the form

(28) $${\dot{x}}_{i}\left(t\right)=f_{i}+\sum_{j=1,j\neq i}^{D}\left(\alpha_{\mathrm{ij}}\sin\left[x_{i}\left(t\right)-x_{j}\left(t\right)\right]+\beta_{\mathrm{ij}}\cos\left[x_{i}\left(t\right)-x_{j}\left(t\right)\right]\right)$$

where the model parameters comprise the parameters *f*, *α* and *β*. A model with *D* states therefore has *P* = *2D^2^* − *D* parameters. We used a cost function equal to the mean square deviation between observed and predicted state trajectories i.e., the norm of the prediction error (again, all states were observed).

**Fig. 4:**
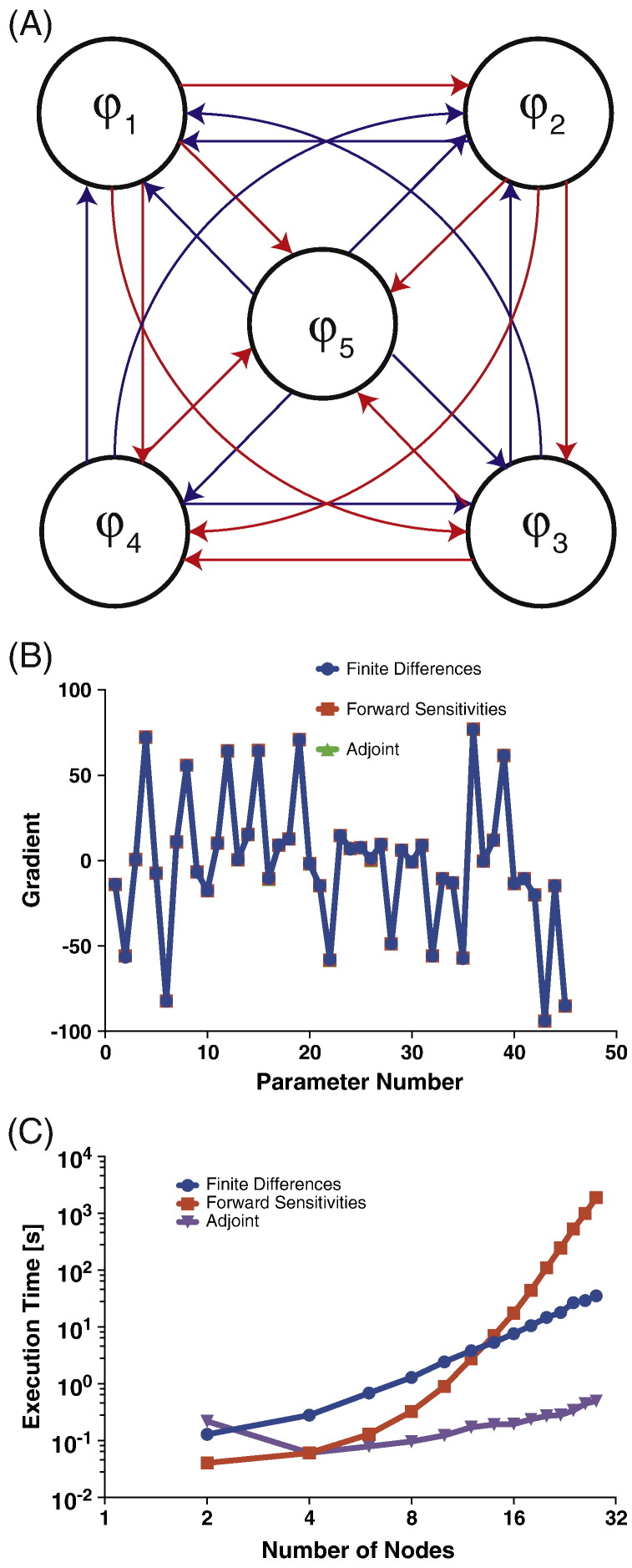
Computational efficiency for non-linear systems (A) Comparison of the gradient obtained by the three methods. Here, the last five parameters quantify the intrinsic oscillator frequencies, and the first 40 parameters the sine and cosine interaction terms. (B) Scaling of run-time as a function of the number of nodes. The absolute and relative tolerances of FD and FS methods were set to 10^− 7^ while the tolerances for the AM method were fixed to 10^− 3^. Simulation time was fixed at 100 ms.

The tolerance parameters of the integration process were set identical to those used for the linear models. Again, the adjoint equation being a linear first order ODE enables the use of lower tolerances (10^− 3^). This process was implemented for a *D* = 5 dimensional problem and Fig. 4(B) shows the estimated gradients.

We then compared the three gradient computation methods. Fig. 4(C) plots the computation time as a function of state dimension. For a 24-node system with 1128 model parameters the computation time for the adjoint method is 50 times less than for the finite difference method.

The efficiency of the AM formulation is due to two reasons — first, the adjoint equation is linear and second it is integrated only once to compute the gradient. Given that the AM equation is linear, the condition number is low, enabling any ODE integrator to integrate the adjoint equation with ease. Indeed, if the ODE integrator is subjected to unnecessary high tolerances it spends more time integrating the adjoint equation. Thus, the advantage of the adjoint scheme reveals both the parsimonious integration scheme as well as the linearity of this equation that requires less-conservative tolerances.

## Discussion

Optimization theory attaches mathematical well-posedness to the issue of biological data-assimilation by formalizing the relationship between empirically measured data and a model generating those responses. In this paper, we introduced three different methods for numerical gradient estimation that forms an integral part of any convex optimization framework. Our comparison establishes that the adjoint method is computationally more efficient for numerical estimation of parametric gradients for state-space models — both linear and non-linear, as in the case of a dynamical causal model (DCM). As is apparent from the gradient equations, the adjoint method is efficient when the numbers of parameters are much greater than the number of states determining the cost function. The contrary is true for the Forward Sensitivity approach albeit for large state-space models, finite-difference based gradients prove to be beneficial. There are two remarks that can be made about the adjoint formulation. First, regardless of whether the flow is linear or non-linear the adjoint method requires the integration of a single linear equation — the computational efficiency is inherent in the structure of the equation. Second, the appearance of a transpose on the adjoint vector implies that the flow of information in the system of equations is reversed; it is in this sense that the adjoint equations are integrated backwards in time.

Although, adaptive error correction is invariably used in the integration of differential equations, the numerical simulations suggest that the tolerance used for integrating the flow and adjoint differential equations are vital in determining the accuracy of the parametric gradients, due to the presence of discretization error. In theory, plugging in the solution field to the flow equation should yield zero, but due to the existence of discretization error the residual is generally non-zero. The same is true for the adjoint equation. In fact, a theorem by Becker and Rannacher (2001) shows how discretization of the gradient depends on the average of the errors and the residuals accumulated in the integration of flow and the adjoint equations (Bangerth and Rannacher, 2003). This is also the case for obtaining gradients via finite-differencing, where we find that the fidelity of error-free discretization of the flow equations is a prerequisite for guaranteeing parametric gradients that are a reliable estimate of the true gradient.

It is known that if the flows are prescribed as ODEs the numerical stability of the adjoint system is guaranteed when the adjoint equation is integrated backwards in time, in the sense that the flow is reversed. Our derivation of the adjoint method is mathematically informal so as to illustrate the basic working principle; rigorous mathematical proofs that accommodate higher order differential algebraic equations, time-dependent parameters or objective functionals that depend on initial conditions are available elsewhere (Cao et al., 2003).

For DCM inversions that allow problem specification in a pre-defined form it may be generally time-efficient to derive the gradient functions analytically rather than using automatic differentiation (Bischof et al., 2012). Automatic differentiation is particularly important for partial differential equations (PDEs) that have 3-dimensional representations, requiring automatization and therefore proving to be error resilient (Sala et al., 2004). For a PDE-constrained optimization problem the solution is governed by a fully coupled Karush–Kuhn–Tucker (KKT) system of equations. These can be computationally expensive for parabolic and hyperbolic PDEs, as well as displaying slow convergence of the defined objective functional (ill-conditioning). The adjoint formulation remedies this by decoupling the coupled PDEs and replacing them by iterative solves of a linear adjoint PDE equation. Additional success of adjoint-based gradient methods for PDE-constrained optimization relies on the fact that mesh independent convergence can be attained. Further speedup could also be obtained by using compiled implementation of forward and adjoint sensitivity methods available in the SUNDIALS time integration package (Hindmarsh and Serban, 2002). This code is written in C and may offer substantial speed advantages over MATLAB implementations.

For data assimilation, it is only rarely that we have precise information on the states or the parameters (Wiener, 1964). Is the adjoint method equally efficient when there is noise on the states and the parameters? One way to represent uncertainty in a mathematical model, whether static or dynamic is to formulate it as a polynomial chaos expansion (Wiener, 1938), one for each noisy state or parameter. This then enables the characteristic statistical quantities to be evaluated as some function of the expansion coefficients — the uncertainty now becomes parameterized. The estimation of the numerical gradient can then proceed akin to a deterministic dynamical model where the computational burden does not depend on the number of parameters (Alekseev et al., 2010). Alternatively, adjoint methods can be gracefully combined with Markov Chain Monte Carlo (MCMC) sampling-based evaluation of the posterior densities (Ghate and Giles, 2005). In a forthcoming paper we address how second-order adjoined gradient estimates could be obtained in the context of Bayesian inversion of neural masses, mean fields, and neural field equations.

Constrained optimization problems arise in many scientific fields, from neuroscience to financial mathematics, therefore a fundamental need for efficient computational methodologies arises. Our work promotes such an endeavor especially for data-sets arising in neuroscience, for example the inversion of large-scale DCMs that have been routinely used to test hypotheses about different functional brain architectures.
